# Macrolide Allergic Reactions

**DOI:** 10.3390/pharmacy7030135

**Published:** 2019-09-18

**Authors:** Kristy M. Shaeer, Elias B. Chahine, Sheeba Varghese Gupta, Jonathan C. Cho

**Affiliations:** 1 Department of Pharmacotherapeutics and Clinical Research, University of South Florida College of Pharmacy, Tampa, FL 33612, USA; 2 Department of Pharmacy Practice, Lloyd L. Gregory School of Pharmacy, Palm Beach Atlantic University, West Palm Beach, FL 33416, USA; elias_chahine@pba.edu; 3 Department of Pharmaceutical Sciences, University of South Florida College of Pharmacy, Tampa, FL 33612, USA; svarghes@health.usf.edu; 4 Department of Clinical Sciences, Ben and Maytee Fisch College of Pharmacy, University of Texas at Tyler, Tyler, TX 75799, USA; jcho@uttyler.edu

**Keywords:** macrolides, allergy, azithromycin, erythromycin, clarithromycin, fidaxomicin, desensitization

## Abstract

Macrolides are antimicrobial agents that can be used to treat a variety of infections. Allergic reactions to macrolides occur infrequently but can include minor to severe cutaneous reactions as well as systemic life-threatening reactions such as anaphylaxis. Most reports of allergic reactions occurred in patients without prior exposure to a macrolide. Cross-reactivity among macrolides may occur due to the similarities in their chemical structures; however, some published literature indicates that some patients can tolerate a different macrolide. Most published reports detailed an allergic reaction to erythromycin. Desensitization protocols to clarithromycin and azithromycin have been described in the literature. The purpose of this article is to summarize macrolide-associated allergic reactions reported in published literature. An extensive literature search was conducted to identify publications linking macrolides to hypersensitivity reactions.

## 1. Introduction

One of the most common causes of medication allergies among adults and children is antibiotics [1]. These allergic reactions can range from immediate to non-immediate (delayed) hypersensitivity reactions. Immediate reactions are typically IgE-mediated and can cause clinical manifestations that include urticaria, angioedema, and anaphylaxis [1,2]. Non-immediate reactions are frequently T-cell mediated and can lead to various degrees of cutaneous symptoms in patients [1,2]. Although many of the reported antibiotic allergies are from the beta-lactam class, cases of allergic reactions to macrolide antibiotics have been documented [1,2,3,4]. However, hypersensitivity reactions resulting from macrolide use occurs infrequently (0.4–3%) [4]. Since macrolide allergies are uncommon, there is a lack of recent data reviewing macrolide allergies and management of those allergies. This paper will review the medicinal chemistry, indications, reported allergic reactions, and desensitization protocols associated with macrolide antimicrobials.

## 2. Medicinal Chemistry

### 2.1. Macrolide Structure

The structure of macrolides consists of a large lactone ring that varies in size from 12 to 18 atoms. Sugar molecules are attached to the lactone ring with glycosidic bonds. Macrolide antibiotics are classified based on the number of atoms in the lactone ring; 14-membered lactones (erythromycin and clarithromycin), 15-membered lactones (azithromycin), ketolide (telithromycin) and 18-membered lactone (fidaxomicin). All clinically available macrolides, with the exception of erythromycin and fidaxomicin, are either synthetically or semi-synthetically generated. Natural macrolides have instability in the gastric environment, resulting in undesired pharmacokinetic properties, such as incomplete absorption resulting in decreased bioavailability [5].

### 2.2. Mechanism of Action

All macrolides except for fidaxomicin block bacterial protein synthesis by binding reversibly to the 23S ribosomal RNA (rRNA) in the 50S-subunit of prokaryotic ribosomes [6]. Fidaxomicin exerts its bactericidal effects by inhibiting bacterial RNA polymerase at transcription initiation by binding to the DNA template-RNA polymerase complex [7].

### 2.3. Structural Aspects behind Cross-Reactivity

**Figure 1:**
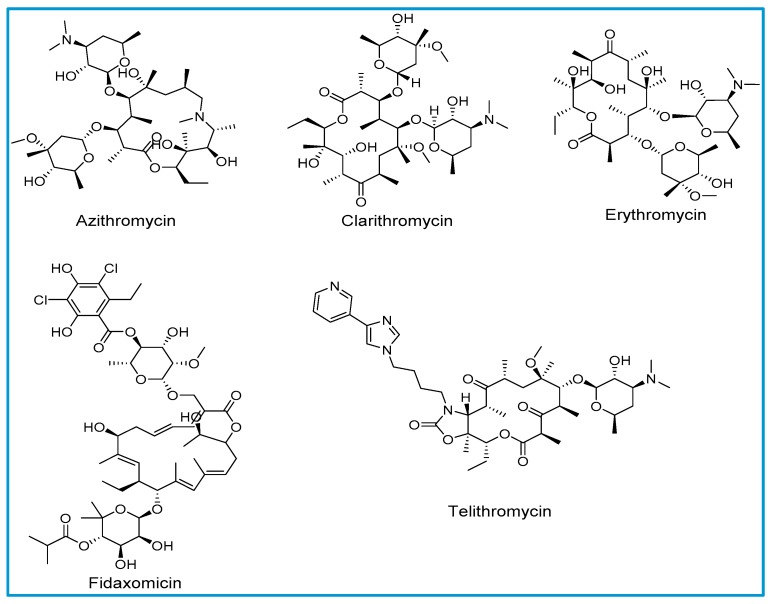
Chemical structures of macrolides.

Allergic reactions to macrolides are relatively less common compared to other classes of antibiotics (Figure 1) [4]. Macrolides with a 14- membered lactone ring such as erythromycin and clarithromycin have been reported to express cross-reactivity in single case reports. The exact mechanism of hypersensitivity due to macrolides is not clearly understood [8]. Azithromycin is a semisynthetic derivative of erythromycin with a 15-membered lactone ring in its structure. Owing to azithromycin’s structural similarity to erythromycin, cross-reactivity with erythromycin has also been reported. There is a lack of scientific evidence to support cross sensitization between various macrolide derivatives [8].

## 3. Place in Therapy

Macrolides are a well-established class of antibiotics. They exhibit bacteriostatic activity against a wide range of gram-positive, gram-negative, and atypical bacteria [9]. Erythromycin, the macrolide with the longest use in practice, has a few remaining indications as the drug of choice given the rise in antibiotic resistance and the availability of more effective and safer antibiotics. Azithromycin and clarithromycin have largely replaced erythromycin in clinical practice because of their broader spectrum of activity, better pharmacokinetics profile, and fewer gastrointestinal adverse effects [9]. In addition, azithromycin is associated with fewer drug–drug interactions than erythromycin and clarithromycin [9].

Macrolides are the drugs of choice for the treatment of various atypical bacteria [9]. Erythromycin is the drug of choice for the treatment of diphtheria caused by *Corynebacterium diphtheriae*, although antitoxin is the primary treatment. Erythromycin is also the drug of choice for the treatment of infants with pneumonia caused by *Chlamydia trachomatis*. Azithromycin is the drug of choice for the treatment of trachoma, urethritis, and cervicitis caused by *Chlamydia trachomatis* and chancroid caused by *Haemophilus ducreyi*. Azithromycin with or without rifampin is also one of the regimens of choice for the treatment of *Legionella pneumophila*, which can cause serious atypical pneumonia. Clarithromycin is the drug of choice for the treatment of *Helicobacter pylori* as part of a combination regimen with amoxicillin and omeprazole. Clarithromycin in addition to amikacin is the regimen of choice for the treatment of infections caused by *Mycobacterium fortuitum* and *Mycobacterium chelonae*. Erythromycin and azithromycin are the drugs of choice for the treatment of infections caused by *Bartonella henselae* (cat scratch fever), *Bartonella quintana* (trench fever), *Campylobacter jejuni* (diarrhea), *Chlamydia trachomatis* (conjunctivitis and urethritis), and *Ureaplasma urealyticum* (urethritis). Azithromycin and clarithromycin are the drugs of choice for the treatment of infections caused by *Mycobacterium avium complex* as part of a combination regimen with ethambutol and rifabutin or monotherapy for primary or secondary prophylaxis. Lastly, all three macrolides are the drugs of choice for the treatment of pertussis (whooping cough) caused by *Bordetella pertussis* and atypical pneumonia caused by *Chlamydophila pneumonia* and *Mycoplasma pneumoniae*.

Many clinical practice guidelines recommend the use of macrolides for the empiric treatment of respiratory tract infections although antibiotic resistance among *Streptococcus pneumoniae*, *Haemophilus influenzae*, and *Moraxella catarrhalis* is on the rise [10,11,12,13]. Current guidelines recommend the empiric use of macrolides as the drugs of choice for the treatment of atypical pneumonia in children and community-acquired pneumonia in adults as monotherapy in the outpatient setting and as combination therapy with a beta-lactam in the inpatient setting [10,11]. Current guidelines recommend the empiric use of macrolides as alternative choices for the treatment of acute otitis media and streptococcal pharyngitis [12,13].

Macrolides can also be used as alternative options for the treatment of various infections in patients who are not able to take the drugs of choice because of allergic reaction or intolerance [9]. For example, erythromycin can be used as an alternative to cephamycins as part of a combination regimen to prevent infections associated with colorectal surgeries or as an alternative to penicillins for the prevention of rheumatic fever. In rare circumstances, erythromycin can be used as an alternative to ciprofloxacin, doxycycline, and penicillins for the treatment of anthrax caused by *Bacillus anthracis* or as an alternative to tetracyclines for the treatment of infections caused by Lymphogranuloma venereum. More commonly, erythromycin can be used as an alternative to tetracyclines for the treatment of acne vulgaris. Azithromycin can be used as an alternative to ceftriaxone or fluoroquinolones for the treatment of typhoid fever caused by *Salmonella typhi* or as an alternative to fluoroquinolones for the treatment of diarrhea caused by *Shigella dysenteriae.* Azithromycin can also be used as an alternative to doxycycline and penicllins for the treatment of Lyme disease caused by *Borrelia burgdorferi* or as an alternative to clindamycin and quinine for the treatment of babesiois caused by *Babesia microti*. Azithromycin and clarithromycin can be used as an alternative to trimethoprim/sulfamethoxazole for the treatment of respiratory infections caused by *Haemophilus influenzae*. Lastly, all three macrolides can be used as an alternative to penicillins for the treatment of respiratory and skin and soft tissue infections caused by groups A, C, and G *Streptococcus, Streptococcus pneumoniae*, and *Moraxella catarrhalis*.

Fidaxomicin is a unique antibiotic, and represents the latest addition to the macrolides [14]. It exhibits bactericidal activity against *Clostridoides difficile*. Fidaxomicin is not systemically absorbed, is well tolerated, and is not associated with any known drug interactions. Clinical trials have shown that fidaxomicin is non-inferior to vancomycin for the treatment of *Clostridioides difficile* infection (CDI) and is associated with lower recurrence rates. Current CDI guidelines recommend the use of fidaxomicin for the treatment of initial severe, non-severe, and recurrent episodes [15].

## 4. Published Allergic Reactions

To gather relevant information, a literature search was performed using the PubMed, EBSCOhost, and Google Scholar electronic databases for articles published up to 17 May 2019, with restrictions for English language and human subjects. Search terms used to identify the included articles were macrolides, azithromycin, clarithromycin, erythromycin, fidaxomicin, hypersensitivity, allergy, rash, toxic epidermal necrosis, Stevens Johnson Syndrome, fixed drug eruption, maculopapular rash, exanthema, and desensitization. Articles about macrolides used as immunosuppressants (e.g., tacrolimus, everolimus, pimecrolimus, and sirolimus) and uncommonly or commercially unavailable were excluded (e.g., kitasamycin, josamycine midecamycin, roxithromycin, spiramycin, telithromycin, and troleandomycin). References of publications for which the full text was retrieved were also reviewed for additional literature sources.

In general, allergic reactions to macrolides reported in the literature are rare. Macrolides are available in a variety of dosage forms, and of those, topical, oral, intravenous, and ophthalmic formulations have been reported to cause an allergic reaction. The initial search for articles regarding macrolide hypersensitivity yielded 1895 citations. Following completion of all search strategies and terms a total of 120 reports were included and summarized in this review. The types of reactions for erythromycin, clarithromycin, azithromycin, and fidaxomicin are summarized in Table 1, Table 2, Table 3 and Table 4. The included reports were published between 1958 and 2018, with reports from 27 different countries. Reported reactions occurred in a variety of patient populations, such as pediatrics (n = 50), adults (n = 105), and unknown (n = 20). Many providers tested patients to confirm the hypersensitivity (n = 79). Several of the reactions involved patients who had received a prior macrolide (n = 43) and of those 23 patients with repeated reactions. Repeated occupational exposures led to 10 subjects with cutaneous adverse reactions to azithromycin [16,17,18].

The breakdown of studies included 88 case reports, 12 case series, 2 cross-sectional, 1 double-blind, placebo-controlled, 5 prospective, and 12 retrospective. There were 58 publications describing erythromycin-associated allergic reactions [19,20,21,22,23,24,25,26,27,28,29,30,31,32,33,34,35,36,37,38,39,40,41,42,43,44,45,46,47,48,49,50,51,52,53,54,55,56,57,58,59,60,61,62,63,64,65,66,67,68,69,70,71,72,73,74,75,76,77]. There were 33 publications describing clarithromycin as a culprit for allergic reactions [72,73,75,78,79,80,81,82,83,84,85,86,87,88,89,90,91,92,93,94,95,96,97,98,99,100,101,102,103,104,105,106]. There were 31 published reports describing azithromycin associated allergic reactions [16,17,18,95,104,107,108,109,110,111,112,113,114,115,116,117,118,119,120,121,122,123,124,125,126,127,128,129,130,131]. Uniquely, the ophthalmic formulation of azithromycin was associated with contact dermatitis [119,123]. There was a single publication dedicated to a case series describing 12 patients who had hypersensitivity reactions to fidaxomicin [132]. Maculopapular exanthema eruptions developed in four subjects suffering from mononucleosis who were also on macrolide therapy [43,109,111,122]. Five cases involved the development of contact dermatitis with topical erythromycin [26,35,51,56,58]. Four cases resulted in a fatality secondary to the severe allergic reaction [19,33,88,91]. Fourteen patients were reported to have anaphylactic reactions to a macrolide [19,23,59,66,73,74,81,98,100,108,121,132]. Drug reaction with eosinophilia and systemic symptoms syndrome was reported in four patients who received azithromycin and one patient who received clarithromycin [105,118,120,124,129]. Erythromycin (n = 1), clarithromycin (n = 2), and azithromycin (n = 1) have been implicated in leukocytoclastic vasculitis [60,79,84,113]. Fourteen publications implicated a macrolide as the cause of an allergic reaction with subjects who received concomitant antimicrobials [25,41,44,45,46,58,76,78,90,101,102,105,119,131]. The most common concomitant agents were an aminoglycoside (n = 3), a beta-lactam (n = 3), and a sulfa (n = 4) [41,44,45,46,78,90,101,102,119,131]. It is possible that one of the concomitant antimicrobials could have elicited the allergic reaction. Two publications excluded a concomitant antimicrobial as the cause with allergy testing [45,58].

Macrolide allergies are rare and available desensitization protocols are restricted to case reports, which all demonstrated success [74,100,102]. Three cases involved successful desensitization with clarithromycin, and one of the patients also completed a desensitization for azithromycin desensitization. Four desensitization protocols were identified in the literature: two involving clarithromycin in an adult patient, one for clarithromycin in a pediatric patient, and one for azithromycin in a pediatric patient (Appendix A, Table A1, Table A2, Table A3 and Table A4) [74,100,102]. Holmes et al. reported a case of a 68-year-old female with a history of anaphylaxis with erythromycin and bronchospasms with roxithromycin who underwent oral clarithromycin desensitization for use in a 18 month treatment course for a skin infection caused by *Mycobacterium chelonae* (Appendix A, Table A1) [74]. Swamy et al. reported a case of a 68-year-old female with a history of anaphylaxis with azithromycin and urticaria and bronchospasms with clarithromycin who underwent oral clarithromycin desensitization for use in a 3 month treatment course for an infection caused by *Mycobacterium intracellularae* (Appendix A, Table A2) [100]. Repeated intradermal skin testing with azithromycin 1 h after desensitization demonstrated a similar reaction seen at baseline. Six weeks after desensitization, azithromycin and clarithromycin skin prick tests were negative and intradermal skin tests were equivocal to the reactions seen at baseline. Petitto et al. described a case of an 11-year-old female with a history of diffuse urticaria with clarithromycin who underwent successful macrolide desensitization for use in osteomyelitis caused by *Mycobacterium fortuitum* (Appendix A, Table A3 and Table A4) [102]. Initially, this patient completed an azithromycin desensitization protocol without complication; however, 24 h later, she developed a generalized urticarial rash within 75 min of a treatment dose. One week later, this patient underwent oral clarithromycin desensitization and all subsequent treatment doses were tolerated.

## 5. Conclusions

Macrolides are infrequently reported to cause various types of allergic reactions, with cutaneous reactions being the most common. Macrolides are similar in chemical structure, and limited reports have demonstrated cross-reactivity. Strategies to overcome hypersensitivity reactions, such as desensitization, or allergy testing for cross-reactivity to another macrolide have been utilized with successful outcomes. Consequently, if a patient has a severe hypersensitivity reaction to a macrolide, then the benefit versus the risk must be evaluated for using an agent in this class. In clinical practice, it may be more convenient and safer to change to an alternative class of medications if the option is available. However, if a macrolide must be used in a patient with a confirmed history of a severe allergic reaction, then allergy testing should be employed to investigate if the patient reacts to other agents in the macrolide class. If so, a desensitization may be conducted while the patient is monitored closely for signs and symptoms of an allergic reaction. 

## Figures and Tables

**Table 1:** Summary of published literature reporting erythromycin hypersensitivity.

Reaction	Time of Onset ^#^	Demographics	Dosage Form	Concomitant Agents	Allergy Evaluation/Confirmation	Prior Sensitization	Notes
Anaphylaxis [19]	5 min	2 y/o F	IM	N	N	N	Fatal
Urticaria [20]	1 day	24 y/o M	IM	N	Y (IDT+)	N	
MPR [21]	5 days	NR (< 18 y/o)	PO	N	Y (DPT+)	N	
MPR [21]	2 days	NR (< 18 y/o)	PO	N	N	N	
MPR [21]	NR	NR (< 18 y/o)	PO	N	Y (DPT−)	N	
MPR [22]	NR	NR	PO	N	N	N	
Anaphylaxis (dyspnea, laryngeal edema) [23]	Several h	60 y/o M	PO	N	Y (MDT+)	N	
HSP [24]	1–1.5 days	51 y/o M	PO	N	N	N	
FDE [25]	12+ h *	22 y/o M	PO	Y (terramycin)	N	Y (E, with same reaction)	
Urticaria [26]	7 days	7 y/o F	PO	N	Y (PT−, ST+)	N	
CD [26]	21 days	52 y/o F	TOP	N	Y (PT+)	N	
Cholestatic hepatitis [27]	1.5 days	53 y/o F	PO	N	Y (LTT+)	Y (E, 2.5 yrs prior no reaction)	
MPR [28] Hepatotoxicity	1 day	7 y/o M	PO	N	N	Y (28 days prior no reaction)	
Rash [29]	8 days	51 y/o M	IV	N	N	N	
Rash [30]	7 days	18 y/o F	PO	N	Y (DPT+)	N	
Cholestatic hepatitis [31]	17 days	15 y/o F	PO	Y (propoxyphene)	N	N	
Cholestatic hepatitis [31]	7 days	23 y/o M	PO	N	N	N	
Cholestatic hepatitis [31]	1 day	44 y/o F	PO	Y (propoxyphene)	N	Y (E, 6 mo prior)	
Cholestatic hepatitis [31]	NR	35 y/o F	PO	N	N	N	
Cholestatic hepatitis [31]	49 days	17 y/o F	PO	N	N	N	
Cholestatic hepatitis [31]	7 days	54 y/o F	PO	N	N	N	
Cholestatic hepatitis [31]	1 day	22 y/o F	IV, PO	N	N	N	
Cholestatic hepatitis [31]	NR	19 y/o M	PO	N	N	Y (E, unknown time)	
Cholestatic hepatitis [31]	7 days	13 y/o F	PO	N	N	N	
MPR [32]	8 days	59 y/o F	PO	N	N	N	
SJS [33]	7days	8 y/o M	PO	N	N	N	Fatal
Hepatotoxicity [34]	Several h	23 y/o M	PO	N	N	Y (E, 13 yrs prior)	
Hepatotoxicity [34]	Several h	71 y/o F	PO	N	N	Y (E, 15 yrs prior)	
CD [35]	NR	72 y/o F	TOP	N	Y (PT+)	Y (E, 2 yrs prior)	
Interstitial Nephritis [36]	Several wks	39 y/o F	PO	N	Y (MMIT+)	N	
Rash [37]	9 days	NR	PO	N	N	N	
Cholestatic hepatitis [38]	2 days	46 y/o F	PO	N	N	Y (E, twice in last 2 mo.)	
Cholestatic hepatitis [38]	6 days	53 y/o F	PO	N	N	Y (E, twice in last 2 mo.)	
FDE [39]	3 days	46 y/o M	PO	N	Y (PT−, DPT +)	Y (E, yrs prior)	
TEN [40]	4–5 h	20 y/o F	PO	N	Y (PT+, DPT+)	N	
Granulomatous interstitial nephritis [41]	5 days	47 y/o M	PO	Y (staggered phenylpropanolamine and amoxicillin)	N	N	
Cholestatic hepatitis [42]	14 days	67 y/o W	PO	N	N	N	
MPE [43]	1 day	23 y/o M	PO	N	N	N	Coninfected with mononucleosis
TEN [44]	2 days	4 y/o M	PO	Y (sulfisoxazole)	Y (DPT+)	Y (15 days prior)	Slow aceylator
FDE [45]	NR	46 y/o M	PO	Y (sulfamide, pyrazolone)	Y (E, PT+, DPT+; sulfa DPT−, magnesium dipirona DPT−)	Y (E, unknown time)	
MAS [46]	NR	n = 26, < 18 y/o and gender NR	PO	Y (sulfisoxazole)	Y (PT+, DPT+)	NR	
MPE [47]	5 min	20 y/o F	PO	N	Y (SPT−, PT− DPT+)	Y (same reaction with spiramycin 1 yr prior)	
FDE [48]	2 and 4 days	27 y/o M	PO	N	Y (DPT+)	Y (E, same reaction months apart)	
MPE, pruritus, fever, hepatitis [49]	2 days	38 y/o F	PO	N	N	N	
Urticaria, palmar pruritus [50]	10 min	25 y/o F	PO	N	Y (SPT+ PT−, DPT+, HRT+)	Y(E, same reaction 92 days apart)	
CD [51]	Several days	46 y/o M	TOP	N	Y (PT+)	Y (received top and PO sequentially)	
Generalized dermatitis [51]	< 1 day	46 y/o M	PO	N	Y (PT+)	Y (received top and PO sequentially)	
SJS [52]	16 h	64 y/o M	PO	N	N	N	
Urticaria [53]	30 min	27 y/o F	PO	N	Y (SPT+, DPT+)	Y (same reaction 2 yrs apart)	Serum IgE+
AGEP [54]	2 days	46 y/o F	PO	N	Y (PT+)	Y (S, 2 days apart)	Cross reactivity with E and S
SJS [55]	< 24 h	23 y/o F	PO	Y (chlorpheniramine, pseudoephedrine, naphazoline)	N	Unknown	
CD [56]	8 days	21 y/o F	TOP	N	Y (PT+)	Y (TOP E multiple times without reaction)	
Cholestatic hepatitis [57]	4 days; 5 days	38 y/o F	PO	N	Y (DPT+)	Y (E, same reaction 1.5 yrs apart)	
CD [58]	12 days	35 y/o M	TOP	Y (metronidazole PO and TOP)	Y (E, PT+; metronidazole PT−)	N	
Anaphylaxis [59]	30 min	24 y/o M	PO	N	Y (SPT−, IDT− DPT+)	N	
LCV [60]	Several hours	1.5 y/o M	PO	N	Y (LTT+)	Y (E unknown time)	Declined PT
Hypersensitivity unspecified [61]	NR	N = 5 4 M, 1 F; 3–60 y/o	NR	NR	Y (E, n = 3 SPT+, n = 5 DPT+; C and A SPT− and DPT−)	NR	No cross-reactivity with E, A, C
Pustulosis [62]	4 days	23 y/o M	PO	N	Y (E: SPT−, PT−, DPT+; C: DPT−)	N	Cross reactivity with E and C
Urticaria [63]	NR	NR	PO	NR	Y (IDT+)	NR	
MPR [63]	NR	NR	PO	NR	Y (SPT+)	NR	
FDE [64]	NR	0–90 y/o M or F	NR	NR	Y (n = 6, DPT+)		
Cholestatic hepatitis [65]	10 days	30 y/o F	PO	N	Y (DPT+)	Y (E, same reaction 2 yrs prior)	
Anaphylaxis (Urticaria and angioedema [66]	Several hours	39 y/o M	PO	NR	N	Y (E, unknown time)	
SJS [67]	16–24 h	31 y/o F	PO	N	N	N	
Cutaneous ADR [68]	NR	n = 6, age and gender NR	NR	NR	NR	NR	
SJS [69]	2 days	20 y/o M	NR	Y (APAP, decongestant)	N	NR	
FDE [70]	6 h	64 y/o M	PO	N	Y (E and C, PT+)	Y (E, two prior times with same reaction)	Cross reactivity with E and C
EM, SJS or TEN [71]	NR	n = 4, (<18 y/o and gender NR)	NR	NR	NR	NR	
Urticaria [72]	NR	n = 3, <15 y/o and gender NR	NR	NR	N	NR	
FDE [72]	NR	<15 y/o and gender NR	NR	NR	N	NR	
MPR [72]	NR	<15 y/o and gender NR	NR	NR	N	NR	
MPR [73]	NIR	<18 y/o and gender NR	NR	NR	Y (n = 3, SPT−, DPT−)	NR	
Anaphylaxis [74]	NR	68 y/o F	NR	NR	N	Y (R)	Desensitized (C)
Exanthema [75]	12 h	19 y/o gender NR	PO	N	Y (IDT−, PT−, SPT−, ODT+)	N	
Exanthema [75]	8 h	22 y/o gender NR	PO	N	Y (IDT−, PT−, SPT−, ODT+)	N	
AGEP [76]	3 days	61 y/o M	PO	Y (fluconazole)	N	NR	
Urticaria [77]	NR	n = 1 (≥17 -79 y/o)	NR	NR	Y (DPT+)	NR	

Abbreviations: ACDR = acute cutaneous drug reaction^¤^; ADR = adverse drug reaction; AGEP = acute generalized exanthematous pustulosis; APAP = acetaminophen; ASA = aspirin; A = azithromycin; C = clarithromycin; CD = contact dermatitis; DR = delayed reaction; DPT = drug provocation test; DRESS = Drug reaction with eosinophilia and systemic symptoms syndrome; E = erythromycin; EM = erythema multiforme; ER = extended release; ETH = ethambutol; F = female; FDX = fidaxomicin; HRT = leukocyte histamine release test; HSP = Henoch- Schönlein Purpura; h = hour(s); HCTZ = hydrochlorothiazide; HIV = human immunodeficiency virus; IBU = ibuprofen; IDT = intradermal test; Ig E = immunoglobulin E; IM = intramuscular; IR = immediate reaction (<1 hr); LABD = Linear Immunoglobulin A Bullous Dermatosis; LCV = leukocytoclastic vasculitis; LTT = lymphocyte transformation test; MAS = multiple allergy sensitivity; MDT = mast cell degranulation test; min= minutes; mo = months; MMIT = macrophage migration inhibitory test; MPE= maculopapular exanthema; MPR = maculopapular rash; N = no; NIR= nonimmediate reaction (1–72 h); NR= not reported; NTG = nitroglycerin; opth = ophthalmic; PT = patch test; R = roxithromycin; RIF = rifampin; RFB = rifabutin; S = spiramycin; SJS = Steven’s Johnson syndrome; SOB = shortness of breath; SPT = skin prick test; ST = scratch test; TEN = toxic epidermal necrolysis; TMP/SMX= trimethoprim/sulfamethoxazole; top = topical; wks = weeks; Y = yes; yrs = years; y/o = years old. * Exact time course unknown as awoke with FDE. ^#^ Time of onset means time when symptoms began to occur either during macrolide therapy or after completion of macrolide therapy^.^ ACDR defined as allergic reaction, adverse drug reaction, pruritis, general swelling, local or general redness, erythema, rash, urticaria, or other skin disease. ^π^ Review was meant to include azithromycin, clarithromycin, erythromycin, and fidaxomicin; however, there is a chance these data include excluded macrolides since the type of macrolides reported were not fully specified

**Table 2:** Summary of published literature reporting clarithromycin hypersensitivity.

Reaction	Time of Onset ^#^	Demographics	Dosage Form	Concomitant Agents	Allergy Evaluation/Confirmation	Prior Sensitization	Notes
Urticaria [72]	NR	n = 1, <15 y/o and gender NR	NR	NR	N	NR	
MPR [72]	NR	n = 1, <15 y/o and gender NR	NR	NR	N	NR	
Anaphylaxis (urticaria, angioedema) [73]	NIR	< 18 y/o and gender NR	NR	NR	Y (SPT−, DPT−)	NR	
MPR [73]	NIR	<18 y/o and gender NR	NR	NR	Y (SPT−, DPT−)	NR	
FDE [75]	6 h	37 y/o and gender NR	PO	NR	Y (IDT−, PT−, SPT−, ODT+)	NR	
Exanthema [75]	20 h	50 y/o and gender NR	PO	NR	Y (IDT−, PT−, SPT−, ODT+)	NR	
Thrombocytopenia [78]	2 weeks	30 y/o M	PO	Y (amikacin, clofazimine)	N	N	
LCV [79]	1 day	68 y/o M	PO	N	N	N	
Thrombocytopenic purpura [80]	7 days	74 y/o M	PO	Y (digoxin)	N	N	
Angioedema [81]	2 h	92 y/o F	PO	Y (NTG, ASA, digoxin, captopril)	N	Y (6 days prior)	
MPR and angioedema [82]	8 h	25 y/o F	PO	N	Y (SPT−, PT−, DPT+)	Y (unknown time)	
FDE [83]	3 days	58 y/o M	PO	N	Y (PT+)	N	
LCV [84]	6 days	83 y/o F	PO	Y (diltiazem ER, ASA, triamterene/HCTZ)	N	N	
HSP [85]	10 days	25 y/o M	PO	N	N	N	
FDE [86]	3 days	83 y/o F	PO	Y (carbosistein, salicylamide, APAP, caffeine, promethazine methylenedisalicilate)	Y (PT−, DPT+)	N	
Dyspnea, bronchospasm, cough [87]	Min	44 y/o F	PO	N	Y (DPT+)	N	
Hepatitis and TEN [88]	7 days	47 y/o M	PO	Y (disulfiram, APAP)	N	N	Fatal
HSP [89]	4 days	48 y/o M	PO	N	N	N	
Pulmonary infiltrates [90]	3 days (episode 1 & 3) 12 days (episode 2)	17 y/o M	PO	Y (cefotaxime episode 2, prednisone episode 3)	Y (DPT+)	Y (same reaction 3 times)	
TEN [91]	3 days	65 y/o W	PO	NR	N	N	Fatal
Eosinophilic pneumonia [92]	3 days	74 y/o M	PO	NR	Y (LTT−, DPT +)	N	
FDE [93]	4 days	68 y/o F	PO	NR	Y (PT−, DPT+)	N	
TEN [94]	2 days	29 y/o F	PO	APAP, ASA and erdosteine	N	N	
SJS [95]	NR	<18 y/o and gender NR	PO	IBU	N	N	
TEN [96]	2 days	2 y/o	PO	N	N	N	
ADRs [97]	NR	n = 1 (<18 y/o and gender NR)	NR	NR	Y (NR)	NR	
Anaphylaxis [98]	4 days	4 y/o F	PO	Y (fluticasone/salmeterol)	N	Y (unknown time)	
Urticaria [99]	10 min	(<15 y/o and gender NR)	PO	N	Y (SPT−, IDT+, DPT+)	NR	
Urticaria, angioedema [99]	20 min	(<15 y/o and gender NR)	PO	N	Y (SPT−, IDT+, DPT+)	NR	
MPE, pruritis [99]	3 days	(<15 y/o and gender NR)	PO	N	Y (SPT−, IDT+, DPT+)	NR	
MPE, pruritis [99]	4 days	(<15 y/o and gender NR)	PO	N	Y (SPT−, IDT+, DPT+)	NR	
Urticaria, dyspnea [100]	6 days	68 y/o F	PO	N	Y (SPT−, IDT+)	Y (C, 6 mo. prior without reaction)	Desensitized with C
SJS [101]	3–4 days	>18 y/o and gender NR	PO	Y (RFB, TMP/SMX, Hidup herbal tea)	N	Unknown	
SJS [101]	NR	>18 y/o and gender NR	PO	Y (dapsone, ciprofloxacin)	N	Unknown	
Diffuse urticarial rash [102]	15–30 min	11 y/o F	IV	Y (amikacin)	Y (C, SPT−, IDT−, DPT+; A, SPT−, IDT−)	N	Cross reactivity with C and A. Desensitized successfully with C and unsuccessfully A
FDE [103]	NR	30 y/o M	PO	N	Y (DPT+)	Y (same reaction 4 mo. apart)	
IR [104]	<1 hr	n = 37, NR	PO	NR	Y (n = 2 ST+)	NR	
NIR [104]	1–3 h	n = 37, NR	PO	NR	Y (n = 2 ST+, n = 2 DPT+)	NR	
DRESS [105]	4 weeks	79 y/o M	PO	Y (RIF, ETH, levothyroxine, hydrocortisone, tramadol, lisinopril, APAP, carvedilol)	N	NR	
MPE [106]	NR	14 y/o M	PO	NR	Y (SPT−, IDT−, DPT+)	Y (1 mo. apart and same reaction)	
Urticaria [106]	NR	30 y/o F	PO	NR	Y (IDT−, DPT+)	N	
Anaphylaxis [121]	2 days	9 y/o, gender NR	PO	NR	N	N	

Abbreviations: ACDR = acute cutaneous drug reaction^¤^; ADR = adverse drug reaction; AGEP = acute generalized exanthematous pustulosis; APAP = acetaminophen; ASA = aspirin; A = azithromycin; C = clarithromycin; CD = contact dermatitis; DR = delayed reaction; DPT = drug provocation test; DRESS = Drug reaction with eosinophilia and systemic symptoms syndrome; E = erythromycin; EM = erythema multiforme; ER = extended release; ETH = ethambutol; F = female; FDX = fidaxomicin; HRT = leukocyte histamine release test; HSP = Henoch- Schönlein Purpura; h = hour(s); HCTZ = hydrochlorothiazide; HIV = human immunodeficiency virus; IBU = ibuprofen; IDT = intradermal test; Ig E = immunoglobulin E; IM = intramuscular; IR = immediate reaction (<1 hr); LABD = Linear Immunoglobulin A Bullous Dermatosis; LCV = leukocytoclastic vasculitis; LTT = lymphocyte transformation test; MAS = multiple allergy sensitivity; MDT = mast cell degranulation test; min= minutes; mo = months; MMIT = macrophage migration inhibitory test; MPE= maculopapular exanthema; MPR = maculopapular rash; N = no; NIR= nonimmediate reaction (1–72 h); NR= not reported; NTG = nitroglycerin; opth = ophthalmic; PT = patch test; R = roxithromycin; RIF = rifampin; RFB = rifabutin; S = spiramycin; SJS = Steven’s Johnson syndrome; SOB = shortness of breath; SPT = skin prick test; ST = scratch test; TEN = toxic epidermal necrolysis; TMP/SMX= trimethoprim/sulfamethoxazole; top = topical; wks = weeks; Y = yes; yrs = years; y/o = years old. * Exact time course unknown as awoke with FDE. ^#^ Time of onset means time when symptoms began to occur either during macrolide therapy or after completion of macrolide therapy^.^ ACDR defined as allergic reaction, adverse drug reaction, pruritis, general swelling, local or general redness, erythema, rash, urticaria, or other skin disease. ^π^ Review was meant to include azithromycin, clarithromycin, erythromycin, and fidaxomicin; however, there is a chance these data include excluded macrolides since the type of macrolides reported were not fully specified

**Table 3:** Summary of published literature reporting azithromycin hypersensitivity.

Reaction	Time of Onset ^#^	Demographics	Dosage Form	Concomitant Agents	Allergy Evaluation/Confirmation	Prior Sensitization	Notes
TEN [95]	NR	n = 4, <18 y/o and gender NR	PO	Y (IBU)	N	N	
EM [95]	NR	n = 4, <18 y/o and gender NR	PO	Y (IBU)	N	N	
SJS [95]	NR	n = 4, <18 y/o and gender NR	PO	Y (IBU)	N	N	
SJS [95]	NR	<18 y/o and gender NR	PO	N	N	N	
Anaphylaxis [100]	2 h	68 y/o F	PO	N	Y (SPT+, IDT+)	Y (C, 1 day prior urticaria and dyspnea)	Cross reactivity with C and A. Desensitized with C.
IR [104]	<1 h	n = 6, NR	PO	NR	Y (n = 4 ST+)	NR	
NIR [104]	1–3 h	n = 13, NR	PO	NR	Y (n = 5 ST+, 8 ST−)	NR	
Toxic pustuloderma [107]	16 h	34 y/o F	PO	N	N	N	
Angioedema [108]	NR	NR	NR	NR	N	NR	
MPE [109]	7 days	20 y/o M	PO	N	N	N	Coinfected with mononucleosis
AMS, fever, generalized rash, and hepatitis [110]	5 days	79 y/o M	PO	Y (atenolol, benazepril, clonidine, ASA)	N	NR	
MPE [111]	<1 day	19 y/o M	PO	N	N	N	Coinfected with mononucleosis
Mild pruritus [112]	NR	n = 135, NR	PO	NR	N	NR	
Severe pruritus [112]	NR	n = 19, NR	PO	NR	N	NR	
Rash [112]	NR	n = 6, NR	PO	NR	N	NR	
LCV [113]	3 days	8 mo. M	PO	NR	N	NS	
SJS [114]	3 days	5 y/o M	PO	N	N	N	
FDE [16]	6 yrs	n = 2, 40 y/o M and 44 y/o M	TOP	N	Y (PT+)	N	Occupational exposure
EM [115]	NR	NR	NR	NR	N	N	
SJS [116]	10 days	62 y/o F	PO	NR	N	N	
CD [17]	3 mo-4 yrs	>18 y/o, gender NR	TOP	NR	Y (n = 4, PT+)	N	Occupational Exposure
LABD [117]	3 days	54 y/o M	PO	N	N	N	
MPR DRESS Myocarditis [118]	H 7 days 2 mo.	48 y/o M	PO	NR	N	N	Skin and heart biopsy proven
CD [18]	28 days	39 y/o M	TOP	NR	Y (A, PT +; C, PT−)	N	No cross reactivity with A and C. Occupational Exposure
CD [119]	21 days	76 y/o F	OPTH	Y (tobramycin/dexamethasone)	Y (PT+)	N	No cross-reactivity with A and C
DRESS [120]	5 days	8 y/o M	PO	N	N	N	
Anaphylaxis [121]	2 days	8 y/o, gender NR	IV	NR	Y (SPT+, IDT+)	NR	
Anaphylaxis [121]	2 days	9 y/o, gender NR	PO	NR	Y (SPT+, IDT+)	Y (C, 1 yr prior same reaction)	Cross reactivity with A and C.
Anaphylaxis [121]	1 day	7 y/o, gender NR	PO	NR	Y (SPT−, IDT+)	Unknown	
MPE [122]	2 days	23 y/o M	PO	N	N	N	Coinfected mononucleosis
CD [123]	NR	85 y/o F	OPTH	N	Y (A, PT +; C and E PT−)	Y (A, 1 yr prior no reaction)	No cross reactivity with A, C, E.
DRESS [124]	7 days	44 y/o M	PO	Y (promethazine, dextromethorphan)	N	N	
AGEP [125]	2 days	18 y/o F	PO	N	N	N	
FDE [126]	<24 h	35 y/o M	PO	N	Y (DPT+)	Y (A, same reaction ~12 mo. apart)	
SJS [127]	1 day	58 y/o M	PO	Y (atenolol, atorvastatin, famotidine, prednisone, hydroxyzine)	N	N	
AGEP [128]	1 day	71 y/o F	PO	NR	Y (A, PT−, DPT+; E PT−; C PT− and C DPT−)	NR	No cross reactivity with A, C, E.
DRESS [129]	4 days	1.4 yrs M	PO	Y (pranlukast)	N	N	
FDE [130]	1 day	50 y/o F	PO	N		Y (A, same reaction 2 yrs prior)	
SJS [131]	1 day	6 y/o M	PO	Y (cefmetazole)	N	N	

Abbreviations: ACDR = acute cutaneous drug reaction^¤^; ADR = adverse drug reaction; AGEP = acute generalized exanthematous pustulosis; APAP = acetaminophen; ASA = aspirin; A = azithromycin; C = clarithromycin; CD = contact dermatitis; DR = delayed reaction; DPT = drug provocation test; DRESS = Drug reaction with eosinophilia and systemic symptoms syndrome; E = erythromycin; EM = erythema multiforme; ER = extended release; ETH = ethambutol; F = female; FDX = fidaxomicin; HRT = leukocyte histamine release test; HSP = Henoch- Schönlein Purpura; h = hour(s); HCTZ = hydrochlorothiazide; HIV = human immunodeficiency virus; IBU = ibuprofen; IDT = intradermal test; Ig E = immunoglobulin E; IM = intramuscular; IR = immediate reaction (<1 hr); LABD = Linear Immunoglobulin A Bullous Dermatosis; LCV = leukocytoclastic vasculitis; LTT = lymphocyte transformation test; MAS = multiple allergy sensitivity; MDT = mast cell degranulation test; min= minutes; mo = months; MMIT = macrophage migration inhibitory test; MPE= maculopapular exanthema; MPR = maculopapular rash; N = no; NIR= nonimmediate reaction (1–72 h); NR= not reported; NTG = nitroglycerin; opth = ophthalmic; PT = patch test; R = roxithromycin; RIF = rifampin; RFB = rifabutin; S = spiramycin; SJS = Steven’s Johnson syndrome; SOB = shortness of breath; SPT = skin prick test; ST = scratch test; TEN = toxic epidermal necrolysis; TMP/SMX= trimethoprim/sulfamethoxazole; top = topical; wks = weeks; Y = yes; yrs = years; y/o = years old. * Exact time course unknown as awoke with FDE. ^#^ Time of onset means time when symptoms began to occur either during macrolide therapy or after completion of macrolide therapy^.^ ACDR defined as allergic reaction, adverse drug reaction, pruritis, general swelling, local or general redness, erythema, rash, urticaria, or other skin disease. ^π^ Review was meant to include azithromycin, clarithromycin, erythromycin, and fidaxomicin; however, there is a chance these data include excluded macrolides since the type of macrolides reported were not fully specified

**Table 4:** Summary of published literature reporting fidaxomycin hypersensitivity.

Reaction	Time of Onset ^#^	Demographics	Dosage Form	Concomitant Agents	Allergy Evaluation/Confirmation	Prior Sensitization	Notes
Diffuse rash [132]	5 days	73 y/o M	PO	NR	N	N	
Rash, swollen eyes and lips [132]	1–2 h	74 y/o F	PO	NR	N	N	
Diffuse rash [132]	5 days	26 y/o F	PO	NR	N	N	
Diffuse rash and itching [132]	1 h	49 y/o F	PO	NR	N	Y (E and A allergy)	Cross reactivity with FDX, E, A.
Throat burning [132]	12–24 h	79 y/o F	PO	NR	N	Y (E allergy)	
Throat and chest swelling [132]	2 days; N	F, age NR	PO	NR	Y (DPT+)	Y (F 30 days prior; E allergy)	
Eyes, lips, mouth swelling, itchy face [132]	72 h	53 y/o F	PO	NR	N	N	
Angioedema [132]	5 days, 6 days	56 y/o M	PO	NR	Y (DPT+)	Y (FDX same reaction within 24 h)	
Lip swelling [132]	7 days	F, age NR	PO	NR	N	N	
Severe rash [132]	NR	M, age NR	PO	NR	N	N	
SOB, throat swelling, chest tightness [132]	2 days	50 y/o F	PO	NR	N	N	
BLE edema and rash [132]	5 days	70 y/o M	PO	NR	N	N	
MAS [46]	NR	n = 6, <18 y/o and, gender NR	PO	N	Y (PT+, DPT+)	NR	
ACDR [77]	NR	NR	NR	NR	N	NR	
MAS [133]	NR	n = 34, age and gender NR	NR	NR	N	NR	
+ DPT with ADR [134]	NR	n = 102, age and gender NR	NR	NR	Y (n = 14, DPT+)	NR	
ADRs [97]	NR	n = 4 (<18 y/o), gender NR	NR	NR	Y (NR)	NR	

Abbreviations: ACDR = acute cutaneous drug reaction^¤^; ADR = adverse drug reaction; AGEP = acute generalized exanthematous pustulosis; APAP = acetaminophen; ASA = aspirin; A = azithromycin; C = clarithromycin; CD = contact dermatitis; DR = delayed reaction; DPT = drug provocation test; DRESS = Drug reaction with eosinophilia and systemic symptoms syndrome; E = erythromycin; EM = erythema multiforme; ER = extended release; ETH = ethambutol; F = female; FDX = fidaxomicin; HRT = leukocyte histamine release test; HSP = Henoch- Schönlein Purpura; h = hour(s); HCTZ = hydrochlorothiazide; HIV = human immunodeficiency virus; IBU = ibuprofen; IDT = intradermal test; Ig E = immunoglobulin E; IM = intramuscular; IR = immediate reaction (<1 hr); LABD = Linear Immunoglobulin A Bullous Dermatosis; LCV = leukocytoclastic vasculitis; LTT = lymphocyte transformation test; MAS = multiple allergy sensitivity; MDT = mast cell degranulation test; min= minutes; mo = months; MMIT = macrophage migration inhibitory test; MPE= maculopapular exanthema; MPR = maculopapular rash; N = no; NIR= nonimmediate reaction (1–72 h); NR= not reported; NTG = nitroglycerin; opth = ophthalmic; PT = patch test; R = roxithromycin; RIF = rifampin; RFB = rifabutin; S = spiramycin; SJS = Steven’s Johnson syndrome; SOB = shortness of breath; SPT = skin prick test; ST = scratch test; TEN = toxic epidermal necrolysis; TMP/SMX= trimethoprim/sulfamethoxazole; top = topical; wks = weeks; Y = yes; yrs = years; y/o = years old. * Exact time course unknown as awoke with FDE. ^#^ Time of onset means time when symptoms began to occur either during macrolide therapy or after completion of macrolide therapy^.^ ACDR defined as allergic reaction, adverse drug reaction, pruritis, general swelling, local or general redness, erythema, rash, urticaria, or other skin disease. ^π^ Review was meant to include azithromycin, clarithromycin, erythromycin, and fidaxomicin; however, there is a chance these data include excluded macrolides since the type of macrolides reported were not fully specified.

## Appendix A

**Table A1:** Oral clarithromycin desensitization protocol in an adult [74] *.

Dose No.	Concentration (mg/mL)	Dose	
		mL	mg
1.	0.05	0.1	0.005
2.	0.05	0.2	0.010
3.	0.05	0.4	0.020
4.	0.05	1	0.050
5.	0.05	2	0.100
6.	0.05	4	0.200
7.	0.50	0.8	0.400
8.	0.50	1.6	0.800
9.	0.50	3.2	1.6
10.	0.50	6.4	3.2
11.	5	1.2	6
12.	5	2.4	12
13.	5	4.8	24
14.	50	1	50
15.	50	2	100
16.	50	4	200
17.	50	8	400
18.	50	10	500
Cumulative dose			1298.4

Abbreviations: mg = milligrams; mL = milliliters; no= number. * Serial 10-fold dilutions of a clarithromycin suspension of 125 mg/5 mL (25 mg/mL) were performed to make clarithromycin solutions at 2.5, 0.25, and 0.025 mg/mL. Each dose was administered in 15-min intervals over 4.5 h with close monitoring on the intensive care unit (for 36 h).

**Table A2:** Oral clarithromycin desensitization protocol in an adult [100] *.

Dose No.	Concentration (mg/mL)	Dose	
		mL	mg
1.	0.025	1.25	0.030
2.	0.025	2.50	0.060
3.	0.025	5	0.125
4.	0.250	1	0.250
5.	0.250	2	0.5
6.	0.250	4	1
7.	2.5	0.8	2
8.	2.5	1.6	4
9.	2.5	3.2	8
10.	2.5	6.4	16
11.	25	1.3	32
12.	25	2.5	64
13.	25	5	125
14.	25	10	250
Cumulative dose			503

Abbreviations: mg = milligrams; mL = milliliters; no = number. * Serial 10-fold dilutions of a clarithromycin suspension of 125 mg/5 mL (25 mg/mL) were performed to make clarithromycin solutions at 2.5, 0.25, and 0.025 mg/mL. Each dose was administered in 15-min intervals over 3.5 h.

**Table A3:** Azithromycin oral desensitization protocol in an adolescent [102].

Dose No.	Concentration (mg/mL)	Dose	
		mL	mg
1.	0.025	0.6	0.030
2.	0.025	1.2	0.060
3.	0.025	2.5	0.125
4.	0.250	5	0.250
5.	0.250	1	0.500
6.	0.250	2	1
7.	2.5	4	2
8.	2.5	0.8	4
9.	2.5	1.6	8
10.	2.5	3.2	16
11.	25	6.4	32
12.	25	2.5	64
13.	25	2.5	125
Cumulative dose			253

**Table A4:** Clarithromycin oral desensitization protocol in an adolescent [102].

Dose No.	Concentration (mg/mL)	Dose	
		mL	mg
1.	0.05	0.60	0.030
2.	0.05	1.20	0.060
3.	0.05	2.50	0.125
4.	0.05	5	0.25
5.	0.50	1	0.50
6.	0.50	2	1
7.	0.50	4	2
8.	5	0.8	4
9.	5	1.6	8
10.	5	3.2	16
11.	5	6.4	32
12.	50	2.5	125
13.	50	2.5	125
Cumulative dose			314
